# Impact of Rheumatoid Arthritis on Hospitalized Inflammatory Bowel Disease Patients: Study of Demographics, Clinical Characteristics and Outcomes

**DOI:** 10.7759/cureus.65011

**Published:** 2024-07-20

**Authors:** Sajana Poudel, Manoj Ghimire, Karun Shrestha, Ayusha Poudel, Kalpana Ghimire, Prakriti Subedi, Osna Pandey, Seema Oli, Rishab Khanal

**Affiliations:** 1 Internal Medicine, John H. Stroger, Jr. Hospital of Cook County, Chicago, USA; 2 Internal Medicine, St Barnabas Hospital, Bronx, USA; 3 Internal Medicine, University of Pittsburgh Medical Center, Harrisburg, USA; 4 Pediatrics, Institute of Medicine, Kathmandu, NPL

**Keywords:** inflammation, chronic autoimmune disease, national inpatient sample database, : rheumatoid arthritis, inflammatory bowel disease

## Abstract

Background

Inflammatory bowel disease (IBD), comprising ulcerative colitis and Crohn's disease gives rise to chronic intestinal inflammation. Rheumatoid arthritis (RA) is a chronic autoimmune disorder characterized by joint and systemic inflammation. IBD is often linked with various autoimmune diseases, with RA being one of the most common. The coexistence of IBD and RA results in an increased inflammatory state, significantly compromising quality of life. Understanding the epidemiological and clinical characteristics of IBD patients with RA is essential for optimizing their management and improving outcomes.

Methodology

This retrospective observational study utilized data from the National Inpatient Sample (NIS) database from 2016 to 2020. Patients aged 18 years and older with a primary discharge diagnosis of IBD were included. This population was subdivided into two groups based on the presence and absence of RA. The primary objective was to compare outcomes between hospitalized IBD patients with and without RA. Key outcomes assessed included mortality rates, hospital length of stay (LOS), and total hospital charges. Secondary outcomes included the prevalence of comorbidities and IBD-related complications.

Results

From 2016 to 2020, a total of 455,655 hospitalized IBD patients were identified, among whom 10,590 (2.32%) had an underlying diagnosis of RA. Patients with both IBD and RA were significantly older than those without RA (mean age 52.21 vs. 45.72 years, p < 0.001) and had a higher proportion of females (72.51% vs. 53.27%, p < 0.01). RA patients exhibited a greater risk of cardiovascular risk factors compared to non-RA patients, including diabetes [adjusted odd ratio (aOR ) 1.12 (1.09-1.16)], hypertension [aOR 1.19 (1.07-1.33)], hyperlipidemia [aOR 1.61 (1.60-1.63)], chronic kidney disease stage 1-4 [aOR 1.35 (1.29-1.41)], coronary artery disease [aOR 1.67 (1.65-1.69)], and heart failure [aOR 1.45 (1.43-1.48)]. However, there were no significant differences in the rates of IBD-related complications or in-hospital mortality between the two groups. The mean hospital LOS was 5.15 days for RA patients and 4.95 days for non-RA patients (p = 0.08), with similar total hospital charges ($48,442.7 vs. $48,720.3, p = 0.88).

Conclusion

This study shows hospitalized IBD patients with and without RA have similar hospitalization outcomes, however, patients with RA have a higher cardiovascular risk. The findings emphasize the importance of integrated, multidisciplinary management approaches for these patients, addressing not only their gastrointestinal and rheumatologic conditions but also their associated comorbidities.

## Introduction

Inflammatory bowel disease (IBD) encompasses ulcerative colitis and Crohn's disease, both of which have recurring episodes of inflammation in the intestine [1]. IBD is associated with several extraintestinal manifestations, with arthritis being one of the most common [2]. The incidence and association of other autoimmune diseases with IBD are well known [3]. Rheumatoid arthritis (RA) is a chronic autoimmune disease that has both articular and extra-articular manifestations [4].

IBD and RA involve significant systemic inflammation and are driven by a complex interplay of genetic, environmental, and immune factors. Recent studies have noted that RA tends to occur frequently with IBD and patients with IBD have a higher risk of having RA [5]. Given that both RA and IBD are progressive and disabling inflammatory diseases, their co-occurrence compromises the quality of life and prognosis for these patients.

In our study, we aimed to determine the in-hospital outcomes such as in-hospital mortality, hospital length of stay, cost of hospitalization, and various in-hospital complications in patients with IBD and RA. Additionally, we identified the socio-demographic and clinical characteristics of these patients. 

## Materials and methods

Study population and design 

**Figure 1:**
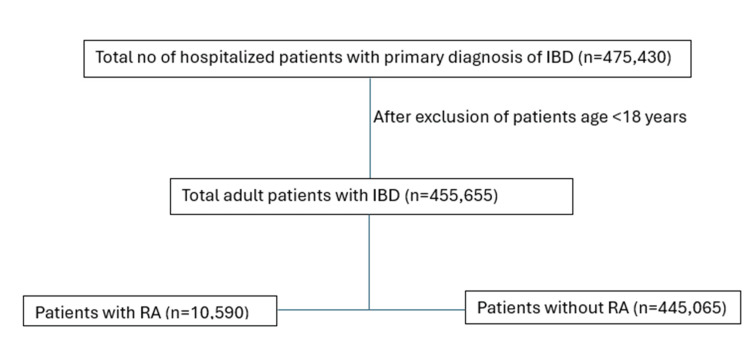
Study flow diagram

We conducted a retrospective, observational study utilizing National Inpatient Sample (NIS) data from 2016-2020. This comprehensive database contains vast inpatient hospital stay information across the United States. All adult patients (age ≥ 18 years) admitted with a primary diagnosis of IBD with or without a secondary diagnosis of RA were included in the study as shown in Figure 1. The data regarding various patient demographics, primary and secondary diagnoses, clinical outcomes, and other relevant patient characteristics were extracted using the International Classification of Diseases, Tenth Revision, and Clinical Modification (ICD-10-CM) codes. The data for this study were obtained from the NIS databases, which are publicly accessible via the Healthcare Cost and Utilization Project (HCUP). The ICD-10-CM codes used for identifying the primary and secondary diagnosis were Crohn's disease of the small intestine (K50), ulcerative (chronic) pancolitis (K51), and rheumatoid arthritis (M05, M06) (Appendix 1).

Primary outcome and variables 

The primary outcome of this study was to compare differences in in-hospital mortality, hospital length of stay, and total hospital charges among IBD patients with and without rheumatoid arthritis (RA). Secondary outcomes included comparing the rates of various IBD-related complications, such as intestinal perforation, sepsis, intestinal obstruction, peritonitis, and gastrointestinal infections, between the two groups.

Statistical analyses

Patient information and data were analyzed using Stata (StataCorp. 2021. Stata Statistical Software: Release 17. College Station, TX: StataCorp LLC). Categorical variables were compared using chi-square tests to assess differences in proportions, and continuous variables were compared using Student’s t-tests to evaluate differences in means. Frequencies, confidence intervals, and p-values were reported for all outcomes. Statistical significance was determined at a p-value < 0.05. 

## Results

From 2016 to 2020, we identified 455,655 patients with a primary discharge diagnosis of IBD. Among these, 10,590 (2.32%) had an underlying diagnosis of RA. The mean age of hospitalized IBD patients was 45.8 years, with IBD patients with RA being slightly older than those without RA (52.21 vs. 45.72 years, p < 0.01). A higher proportion of females was observed in the RA group compared to the non-RA group (72.51% vs. 53.27%, p < 0.01). About 73.28 % of our hospitalized IBD patients were white, followed by 14% black and 8% Hispanics with similar distribution among both groups. The demographic characteristics are shown in Table 1. 

**Table 1:** Demographic characteristics of the study population Bold p-values are statistically significant; ^1 ^t-test; ^2 ^Chi-square test

Patient characteristics	With Rheumatoid Arthritis (n=10,590)	Without Rheumatoid Arthritis (n=445,065)	t-value / χ²-value / F-value	p-value
Age(years), mean, (±SD)	52.21 (±17.28)	45.72 (±18.12)	16.64	<0.001^1^
Age group, n (%)				
18-29 years	1,160 (10.95)	101,786 (22.87)	6,440	<0.001 ^2^
30-49 years	3,630 (34.28)	167,433 (37.62)	3,778	<0.001 ^2^
≥50 years	5,800 (54.77)	175,846 (39.51)	7,700	<0.001 ^2^
Gender, n (%)				
Female	7,675 (72.51)	237,086 (53.27)	11,800	<0.001 ^2^
Race/Ethnicity, n (%)				
White	7,620 (74.05)	326,054 (73.26)	1,380	<0.001 ^2^
Black	1,665 (16.18)	62,264 (13.99)		
Hispanic	640 (6.22)	36,317 (8.16)		
Asian	90 (0.87)	6,542 (1.47)		
Others	80 (0.78)	1,646 (0.37)		
Native American	195 (1.9)	12,150 (2.73)		

IBD patients with RA were found to have a higher risk of cardiovascular conditions. After adjusting for age, sex, race, insurance category, and the Charlson co-morbidity index, these patients were shown to have an increased risk of diabetes [aOR 1.12 confidence interval (CI): 1.09-1.16], hypertension (aOR 1.19 CI: 1.07-1.33), hyperlipidemia (aOR 1.61 CI: 1.60-1.63), chronic kidney disease (aOR 1.35 CI: 1.29-1.41), coronary artery disease (aOR 1.67 CI: 1.65-1.69), and heart failure (aOR 1.45 CI:1.43-1.48). The clinical characteristics of the study population are shown in Tables 2, 3. 

**Table 2:** Clinical characteristics and outcomes of the study population Bold p values are statistically significant, BMI: body mass index, CI: confidence interval, ^1^ t-test, ^2 ^Chi-Square test

Patient Characteristics	With Rheumatoid Arthritis (n=10590)	Without Rheumatoid Arthritis (n=445065)	t-value / χ²-value / F-value	p-value
Co-morbidities, n (%)				
Diabetes Mellitus	855 (8.07)	24,211 (5.44)	10,600	<0.001 ^2^
Hypertension	3,740 (35.32)	107,349 (24.12)	53,900	<0.001 ^2^
Smoking	135 (1.27)	6925 (1.56)	411	0.29 ^2^
Heart failure	555 (5.24)	14,019 (3.15)	11,100	<0.01 ^2^
End-stage renal disease	20 (0.19)	1,602 (0.36)	632	<0.001 ^2^
Chronic kidney disease (stages 1-5)	735 (6.94)	20,250 (4.55)	10,400	<0.001 ^2^
Hyperlipidemia	2,275 (21.48)	62,353 (14.01)	36,400	<0.001 ^2^
Overweight and obesity (BMI ≥25)	1,410 (13.31)	40,055 (9)	17,900	<0.001 ^2^
Coronary artery disease	1,000 (9.44)	24,879 (5.59)	22,000	<0.001 ^2^
Chronic obstructive pulmonary disease	1,005 (9.49)	21,096 (4.74)	38,800	<0.001 ^2^
Alcohol abuse	65 (0.61)	3,750 (0.84)	500	0.25 ^2^
Complications, n (%)				
*C. difficle* infection	205 (1.94)	6,785 (1.52)	888.6	0.13 ^2^
Toxic megacolon	5 (0.05)	240 (0.05)	6.6	0.89 ^2^
Intestinal obstruction	500 (4.72)	221,642 (4.98)	113.3	0.57 ^2^
Peritonitis	30 (0.28)	1,245 (0.28)	0.35	0.97 ^2^
Myocardial infarction	45 (0.42)	1,575 (0.35)	113	0.58 ^2^
Cardiac arrest	210 (1.98)	7,125 (1.6)	731.5	0.18 ^2^
Cardiogenic shock	10 (0.09)	110 (0.02)	1,464	0.05 ^2^
Arrhythmia	75 (0.71)	2,730 (0.61)	116	0.6 ^2^
Stroke	10 (0.09)	400 (0.09)	1.8	0.94 ^2^
Acute kidney Injury	95 (0.9)	2,385 (0.54)	1,912	0.03 ^2^
Mechanical ventilation	60 (0.57)	1,860 (0.42)	417	0.29 ^2^
Septic shock	45 (0.42)	2,100 (0.47)	37.2	0.75 ^2^
Outcomes				
In-hospital mortality, n (%)	55 (0.52)	1,330 (0.3)	1,276	0.06 ^2^
Length of stay (days), mean (95% CI)	5.15 (4.92-5.37)	4.95 (4.95-4.99)	1.72	0.08 ^1^
Total hospital charges, $, mean (95% CI)	48,720 (44981-52458)	48,442 (47489-49396)	0.15	0.88 ^2^

**Table 3:** Risk of various cardiovascular diseases in IBD patients with RA Bold p values are statistically significant, CI: confidence interval, ^1^ Binary logistic regression, ^2 ^Multiple logistic regression

Variables	Unadjusted odds ratio (95% CI)	p-value	Adjusted odds ratio (95% CI)	p-value
Hypertension	1.71 (1.56-1.88)	<0.001 ^1^	1.19 (1.07-1.33)	<0.001 ^2^
Hyperlipidemia	1.67 (1.50-1.87)	<0.001 ^1^	1.61 (1.60-1.63)	<0.001 ^2^
Heart failure	1.69 (1.28-2.24)	<0.001 ^1^	1.45 (1.43-1.48)	<0.001 ^2^
Coronary artery disease	1.76 (1.51-2.04)	<0.001 ^1^	1.67 (1.65-1.69)	<0.001 ^2^
Diabetes	1.52 (1.29-1.79)	<0.001 ^1^	1.12 (1.09-1.16)	<0.001 ^2^
Chronic kidney disease	1.56 (1.31-1.86)	<0.001 ^1^	1.35 (1.29-1.41)	<0.001 ^2^
Obesity	1.55 (1.36-1.77)	<0.001 ^1^	0.95 (0.82-1.09)	0.49 ^2^

In IBD patients with RA, binary logistic regression revealed no increased risk of mortality (OR 1.74, 95% CI: 0.95-3.19). Additionally, there is no significant increase in hospital length of stay (mean length 5.15 days vs 4.95 days, p=0.08) or total hospital charges (mean cost $48,720 vs $48,442, p=0.88). However, there is a significant increase in the risk of acute kidney injury (AKI) (OR 1.68, 95% CI: 1.03-2.72). There is no significant risk of cardiovascular outcomes such as cardiac arrest, myocardial infarction, and septic shock, or intestinal complications such as *Clostridium difficile* infection, toxic megacolon, intestinal obstruction, and peritonitis. The outcome results are shown in Tables 2, 4.

**Table 4:** Risk of various in-hospital complications in IBD patients with RA Bold p-values are statistically significant, AKI: acute kidney injury, ^1^ Binary linear regression, ^2^ Binary logistic regression, IBD: inflammatory bowel disease, RA: rheumatoid arthritis.

Variables	Odds Raio/ Coefficient (95% CI)	p-value
Mortality	1.74 (0.95-3.19)	0.07 ^2^
Length of stay	0.2 (-0.27 to 0.42)	0.08 ^1^
Total hospital charges	277 (-3412 to 3967)	0.88 ^1^
Cardiac arrest	1.1 (0.27-4.5)	0.88 ^2^
Myocardial Infarction	1.56 (0.8-3.4)	0.18 ^2^
Septic shock	0.9 (0.46-1.74)	0.9 ^2^
AKI	1.68 (1.03-2.72)	0.03 ^2^
*Clostridium difficile* infection	1.2 (0.93-1.55)	0.15 ^2^
Toxic megacolon	0.87 (0.12-6.33)	0.89 ^2^
Intestinal obstruction	0.94 (0.77-1.15)	0.57 ^2^
Peritonitis	1.01 (0.45-2.27)	0.97 ^2^

## Discussion

This study highlights the significant differences in demographic and clinical characteristics between IBD patients with and without RA. Our findings show that IBD patients with RA are older, predominantly female, and have a higher prevalence of various comorbidities compared to those without RA. These differences show the complex health profile and the increased healthcare needs of IBD patients who also suffer from RA.

The prevalence of RA in patients with IBD in our study was 2.34%, which is slightly higher than that in the general population. The worldwide prevalence of RA was estimated at 0.24% based on the Global Burden of Disease 2010 Study [6]. Studies have reported the prevalence of RA in the United States to be around 0.5-1% [7,8,9]. Consistent with our study, a systemic review showed a significantly higher risk of RA among patients with IBD [relative risk (RR) = 2.59; 95% CI: 1.93-3.48] [5]. The higher mean age of RA patients in our study is consistent with the known epidemiology of RA, which predominantly affects older individuals [10]. The predominance of females in the RA group (72.51% vs. 53.27% in the non-RA group) aligns with existing literature that reports a higher prevalence of RA among women in the general population [10].

Our study identified that IBD patients with RA experience a substantial burden of cardiovascular risk factors such as diabetes, hypertension, chronic kidney disease, coronary artery disease, and heart failure. These findings are consistent with previous studies, which have shown that RA patients have a high incidence and prevalence of these cardiovascular conditions, as well as increased mortality rates associated with them [11-14]. A large meta-analysis showed that cardiovascular disease mortality is increased by 50% in RA than in the general population [11]. This increased risk is largely attributed to chronic inflammation [14]. Consequently, IBD patients with underlying RA face an elevated risk of cardiovascular morbidity, leading to poorer clinical outcomes. The presence of multiple comorbidities in RA patients may also contribute to the observed higher healthcare utilization and longer hospital stays, although the latter did not reach statistical significance in this study.

Acute kidney injury was notably more prevalent among RA patients. This may be attributable to the increased use of nephrotoxic medications and the higher burden of comorbidities such as hypertension and diabetes in this population. Complications such as toxic megacolon, intestinal obstruction, and myocardial infarction did not show significant differences, suggesting that these complications might be equally managed in both groups and that their occurrence is not markedly influenced by the presence of RA. Although in-hospital complications were higher among IBD patients compared to the general population, the risk has not been studied in patients with both RA and IBD. The lack of significant difference in total hospital charges between RA and non-RA patients suggests that while RA patients may have a more complex health profile, the acute management costs during hospitalization are comparable. This could be due to standardized care protocols for acute IBD management that mitigate cost discrepancies despite differing comorbid profiles.

Our study has several implications for clinical practice. First, it highlights the need for a multidisciplinary approach in managing IBD patients with RA, addressing not only the gastrointestinal and rheumatologic aspects but also the associated comorbidities. Second, the higher prevalence of comorbidities in RA patients emphasizes the importance of regular screening and proactive management of these conditions to improve overall health outcomes. Third, understanding the demographic differences, particularly the older age and female predominance in RA patients, can inform tailored healthcare strategies and patient education efforts.

However, there are limitations to this study. As the NIS is a retrospective database, the accuracy of the diagnosis may be compromised and we have to rely on ICD-10 codes. We could not identify the specific disease-modifying therapies our patients were receiving. The medications used to treat rheumatoid arthritis and IBD could confound the results, impacting the observed outcomes and associations. Moreover, the cross-sectional design restricts our ability to establish causal relationships between RA and the observed differences in comorbidities and complications. Furthermore, the data were obtained from a hospital-based cohort, which might not fully represent the broader IBD population seen as an outpatient. Future longitudinal studies are necessary to investigate causal pathways and evaluate the long-term outcomes of IBD patients co-affected by RA.

## Conclusions

IBD patients with RA present a distinct and more complex health profile characterized by older age, female predominance, and a higher burden of comorbidities like cardiovascular diseases and metabolic conditions. Enhanced awareness among healthcare providers about the higher risk of comorbidities in IBD patients with RA is crucial. It is important to curate a multidisciplinary management approach to address the unique healthcare needs of this population. Comprehensive management strategies might include regular screenings for cardiovascular conditions and patient education to promote lifestyle changes that can reduce inflammation and improve overall health. Longitudinal studies are essential to further investigate the causal relationships and long-term outcomes in IBD patients with RA, ultimately guiding more effective, personalized care for these patients.

## Appendices

Appendix 1 

**Table 5:** ICD 10 codes used to extract the variables CKD: chronic kidney disease, ESRD: end-stage renal disease, AKI: acute kidney injury,

Diagnosis	ICD-10 CM codes/PCS codes
Rheumatoid arthritis	M05, M06
Ulcerative colitis	K51
Corhn’s disease	K50
Hypertension	I10, I15,
Hyperlipidemia	E78.1, E78.2, E78.3, E78.4, E78.5
Heart failure	I50
Diabetes	E10.1, E10.9, E11.1, E11.9, E13.1, E13.9, E14.1, E14.9, E10.2, E10.4, E11.2, E11.4, E13.2, E13.4, E14.2, E14.4
Overweight and obesity	E66
Coronary artery disease	I25.1, I25.2
Myocardial Infarction	I21
CKD	N18.1, N18.2, N18.3, N18.4, N18.5
ESRD	N18.6
Severe sepsis with/without septic shock	R65.21, R65.20
AKI	N17
Cardiac arrest	I46
Cardiogenic shock	R57.0
Clostridium difficile	A04.7
Toxic megacolon	K59.31
Intestinal obstruction	K56.1, K56.2, K56.3, K56.4, K56.5, K56.6
Peritonitis	K65.0, K65.1, K65.8, K65.9
Stroke	I60,I61, I62, I63. I64, I65, I66, I67, I68, I69
Mechanical ventilation	5A1935Z , 5A1945Z ,5A1955Z,
